# Endoscopic Management of Malignant Biliary Stricture

**DOI:** 10.3390/diagnostics10060390

**Published:** 2020-06-10

**Authors:** Robert Dorrell, Swati Pawa, Rishi Pawa

**Affiliations:** 1 Department of Medicine, Wake Forest School of Medicine, Winston-Salem, NC 27157, USA; rdorrell@wakehealth.edu; 2 Department of Medicine, Division of Gastroenterology, Wake Forest School of Medicine, Winston-Salem, NC 27157, USA; spawa@wakehealth.edu

**Keywords:** malignant biliary stricture, hilar stricture, hepatobiliary malignancy, pancreatic cancer, endoscopic management, self-expandable metal stent, radiofrequency ablation, endoscopic ultrasound-guided biliary drainage

## Abstract

A biliary stricture is an area of narrowing in the extrahepatic or intrahepatic biliary system. The majority of biliary strictures are caused by malignancies, particularly cholangiocarcinoma and pancreatic adenocarcinoma. Most malignant biliary strictures are unresectable at diagnosis. Treatment of these diseases historically required surgical procedures, however, the development of endoscopic techniques has provided alternative minimally invasive treatment options to improve patient quality of life and survival with unresectable disease. While endoscopic retrograde cholangiopancreatography with stent placement has been the cornerstone of biliary drainage for decades, cutting edge endoscopic developments, including radiofrequency ablation and endoscopic ultrasound-guided biliary drainage, offer new therapy options to patients that historically have a poor quality of life and a grim prognosis. In this review, we explore the endoscopic techniques that have contributed to revolutionary advancements in the endoscopic management of malignant biliary strictures.

## 1. Introduction

A biliary stricture is an area of stenosis in the extrahepatic or intrahepatic biliary system. It can be the result of either benign or malignant pathologies, but unfortunately, the majority of biliary strictures are malignant (76–85%) at the time of diagnosis [1]. Extensive diagnostic techniques are required to differentiate the stricture as benign or malignant. Once a diagnosis has been obtained, surgical and/or endoscopic management should promptly commence for curative resection or palliation.

Malignant biliary strictures (MBS) can be divided into hilar strictures (involving the left and right hepatic ducts and their confluence) and distal strictures (involving the common bile duct). Hilar malignant strictures can result from cholangiocarcinoma, gallbladder carcinoma, hepatocellular carcinoma, lymphoproliferative disorders, or metastatic disease. Pancreatic adenocarcinoma is the most common cause of distal malignant bile duct stricture, but other causes include cholangiocarcinoma, ampullary carcinoma, lymphoproliferative disorders, and metastatic disease. Patients with MBS typically have a poor quality of life due to obstructive symptoms and poor prognosis. Fortunately, the expanding field of advanced endoscopy continues to provide better techniques for the management of biliary strictures, whether the goal is curative or palliative. This literature review attempts to summarize options for endoscopic management of malignant biliary strictures and to present the innovative endoscopic techniques in this field.

## 2. Endoscopic Management of Resectable Malignant Biliary Stricture

Once the biliary stricture has been identified as malignant through the various modes of diagnostic and endoscopic imaging and tissue sampling, the strictures are evaluated for possible operative intervention. However, only 30% of biliary strictures are resectable at the time of diagnosis [1].

Endoscopic procedures play a role in open surgical planning [2]. For example, because hyperbilirubinemia portends poor surgical outcomes, preoperative biliary drainage (PBD) can be performed before the surgery to mitigate risk. Early studies showed that placement of a common bile duct stent through endoscopic or percutaneous procedures decreased postoperative risks. However, a randomized control trial performed by Van der Gaag et al. showed no significant change in mortality or hospital length of stay for patients who underwent PBD compared to those who did not in this particular study. Additionally, there was a significant increase in the rate of complications postoperatively for patients who underwent PBD [3]. Furthermore, Fang et al. demonstrated no mortality benefit with PBD in a meta-analysis [4]. Coelen et al. performed a recent randomized control trial in patients with resectable perihilar cholangiocarcinoma comparing endoscopic biliary drainage vs. percutaneous transhepatic biliary drainage (PTBD). The study was canceled early due to a significantly higher mortality in patients undergoing PTBD (41%) vs. endoscopic biliary drainage (11%) [5].

PBD should be reserved for patients who have significant debilitating symptoms, including pruritis, cholangitis, and severe organ dysfunction, or for patients scheduled to undergo neoadjuvant chemotherapy. Current guidelines also state that PBD should be considered if bilirubin levels are greater than 250 umol/l. When PBD is performed, endoscopic drainage is preferred due to decreased morbidity and mortality. Additionally, patients should have a four to six week delay between PBD and surgical resection to allow for normalization of hepatic function between interventions [6]. PBD is still a controversial procedure given the contradictory evidence, and additional research is needed to determine the best candidates and protocol for this procedure.

## 3. Endoscopic Management of Unresectable Malignant Biliary Stricture

The vast majority (70%) of MBS are unresectable at presentation. Endoscopic palliative procedures aim to alleviate symptoms. These include ERCP with stent placement, endoscopic ultrasound-guided biliary drainage, and endobiliary radiofrequency ablation [7]. ERCP with stent placement is the primary palliative tool for biliary drainage in the setting of MBS.

Endoscopic stent placement is the preferred first line intervention due to its improved morbidity and mortality compared to its invasive counterpart, surgical bypass. Three meta-analyses were performed, which showed increased 30 day mortality in surgical bypass patients compared to endoscopic stent placement patients (16.3% vs. 9.6%) [8]. Additionally, surgical bypass has been associated with up to 25% postoperative morbidity and mortality. However, surgical bypass does seem to be superior in avoiding recurrent jaundice. The decision to perform endoscopic stent placement versus surgical bypass should be considered carefully, with the risks and benefits of each procedure discussed thoroughly with patients [9,10].

### 3.1. Plastic vs. Metal Stents

Biliary stents include self-expandable metallic stents (SEMS) and plastic stents (PS). SEMS are the primary stent used for MBS. Plastic stents are less commonly used for many reasons. Zorron et al. performed a systematic review, in which they identified 3660 studies comparing SEMS and PS. SEMS had a significantly reduced stent dysfunction (21.6% vs. 46.8%) [11]. Additionally, patients with SEMS had lower reintervention rates and better survival rates. Moole et al. performed a meta-analysis comparing SEMS vs. PS and found similar results as well as better patency periods, lower re-occlusion rates, and lower rates of cholangitis for SEMS [12]. These results have been duplicated in various other studies as well. The role of plastic stents is typically limited to patients with unresectable disease, with a life expectancy less than three months. In cases in which the stent was placed for less than three months, patency, stent dysfunction, and migration were comparable amongst PS and SEMS (Table 1). Additionally, PS are more cost effective for these patients [13]. Overall, the role of PS is limited to patients with a limited life expectancy due to the superior performance of SEMS and greater cost effectiveness in all other patients [14].

**Figure 1:**
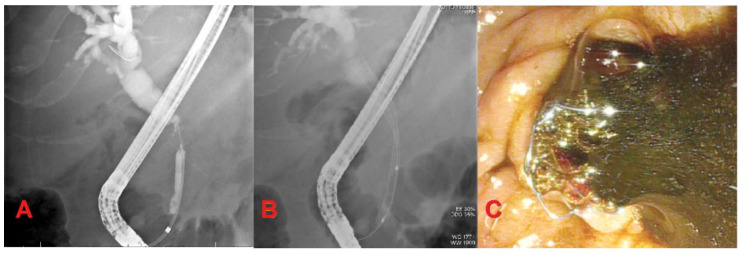
Extrahepatic cholangiocarcinoma. (**A**) Mid-common bile duct stricture with dilation of proximal bile duct and intrahepatics; (**B**) Fully-covered self-expandable metallic stent (FCSEMS) placement for biliary drainage; (**C**) Bile drainage after FCSEMS placement.

Self-expandable metallic stents can be uncovered (UCSEMS), partially covered, or fully-covered (FCSEMS). UCSEMS are less prone to migration, but are difficult to remove due to tissue ingrowth. FCSEMS have a nonporous membranous coating designed to decrease rates of occlusion with easy removability. However, this benefit comes at the expense of increased risk of stent migration [15] (Figure 1).

### 3.2. Unresectable Distal Malignant Biliary Stricture

Distal malignant biliary strictures (DMBS) are most commonly caused by pancreatic adenocarcinoma. Less than 15% of DMBS are resectable at the time of diagnosis; therefore, endoscopic management is classically directed towards palliative therapies, including stenting for biliary drainage and endobiliary ablation [16].

Numerous studies have compared the role of UCSEMS and FCSEMS for management of malignant distal bile duct strictures with conflicting conclusions. Saleem et al. conducted a meta-analysis involving 781 patients comparing FCSEMS to UCSEMS for distal malignant biliary strictures (DMBS). FCSEMS were associated with longer stent patency and stent survival (Table 2) [17]. Conio et al. performed a randomized multicenter trial on 158 patients comparing FCSEMS and UCSEMS for DMBS. They concluded higher stent migration and earlier stent occlusion for FCSEMS [18]. Furthermore, there is a theoretical risk of cholecystitis with FCSEMS. The stent may occlude the cystic duct, hamper gallbladder drainage and place patients at risk for cholecystitis. Majmudar et al. found a 15% increased rate of cholecystitis in FCSEMS vs. UCSEMS [19]. However, these results were not reproducible by Isayama et al., who analyzed 246 patients (171 received FCSEMS and 75 received UCSEMS) and found no statistical difference in rates of cholecystitis amongst patients receiving either stent [20]. Given the lack of consensus in these studies, additional research is needed to determine the role of UCSEMS vs. FCSEMS for DMBS.

### 3.3. Unresectable Hilar Malignant Biliary Stricture (HMBS)

Malignant biliary stricture at the hilum of the liver is most commonly caused by cholangiocarcinoma, local extension from gallbladder and hepatocellular carcinoma, and metastatic cancer. Due to the high rates of unresectability, endoscopic palliation with ERCP and stent placement for biliary drainage and endobiliary radiofrequency ablation are the foremost management options. Endoscopic planning for optimal liver drainage is based on the Bismuth–Corlette classification (Table 3). PS and UCSEMS are the primary stents used in HMBS. FCSEMS are generally avoided in HMBS due to the risk of obstruction of contralateral bile duct and side branch ducts [21].

Palliative treatment for unresectable MBS focuses on relieving symptoms caused by biliary obstruction and hyperbilirubinemia, including pruritus and jaundice. Biliary stenting opens the obstructed ducts to facilitate bile flow and drain liver volume. Two techniques of biliary stenting are often compared: unilateral (the stent is placed in either the right or left hepatic duct) and bilateral (the stent spans both the right and left hepatic duct). There is currently no consensus on whether unilateral or bilateral stenting is more effective at relieving biliary obstruction, as studies have failed to show improved efficacy or lower complication rates for one technique over the other. Meybodi et al. performed a systematic review and meta-analysis comparing unilateral versus bilateral endoscopic stenting [23]. They looked at 21 studies with 1292 patients with biliary strictures. Technical success rates were higher in the unilateral group, which is likely due to the less complex procedure of unilateral stenting. However, functional success of both groups was not significantly different. Additionally, the complication rate was comparable. This study concluded that unilateral and bilateral stenting are analogous in safety and success rates.

**Figure 2:**
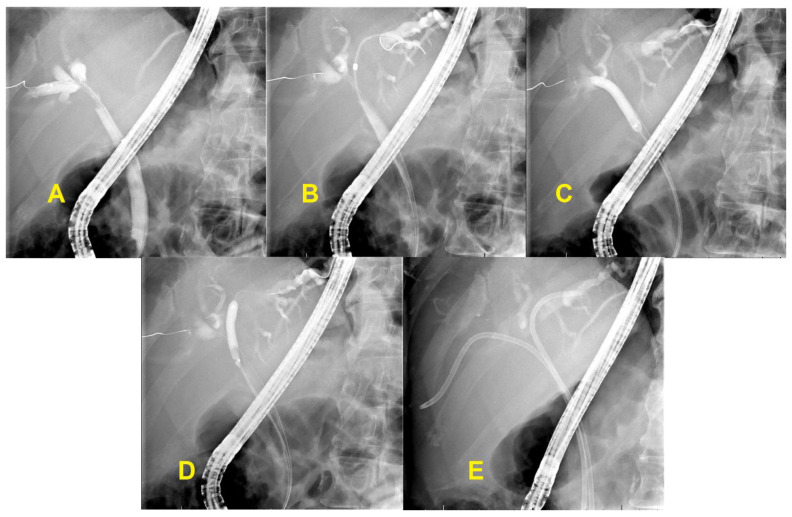
Hilar cholangiocarcinoma. (**A**,**B**) Hilar stricture with dilated right and left intrahepatic biliary tree; (**C**,**D**) Balloon dilatation of hilar stricture; (**E**) Bilateral plastic stents placement.

While the drainage goal for palliation used to be less than 25% of the liver volume, a retrospective study by Vienne et al. demonstrated multiple benefits of increased drainage [24,25]. This study enrolled 107 patients with hilar tumors (bismuth type II–IV) who underwent stent placement and recorded the percentage of biliary drainage after the procedure. Increased biliary drainage (greater than 50% of liver volume) was significantly associated with stent effectiveness (defined as a decrease in bilirubin level of more than 50% of pretreatment bilirubin one month after stent placement), decreased risk of cholangitis, and prolonged survival. Additionally, draining atrophic segments did not improve biliary drainage and increased cholangitis. Based on this study, biliary stenting should aim for at least 50% drainage [24]. Cross-sectional imaging like CT or MRI should be performed prior to stenting to estimate the pre-procedure hepatic volume. Subsequent images can estimate the percentage drained post-procedure and ensure the large volume drainage goals are met (Figure 2).

### 3.4. Endobiliary Radiofrequency Ablation (RFA)

**Figure 3:**
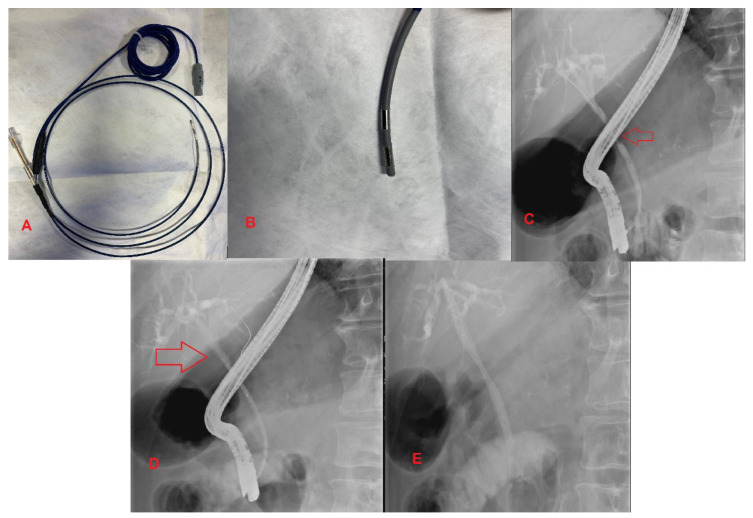
Extrahepatic Cholangiocarcinoma. (**A**,**B**) Habib Endo HPB Bipolar Radiofrequency Catheter; (**C**) Malignant common bile duct stricture; (**D**) The red arrow indicates a radiofrequency ablation catheter in place across a stricture; (**E**) UCSEMS placement status post RFA therapy.

Prior to the development of RFA, endoscopic directed treatment of unresectable MBS consisted of photodynamic therapy (PDT). This procedure involves injection of a photosensitizing chemical with subsequent laser application to the lesion, leading to tissue necrosis. PDT showed promising results; however, there was a significant increase in complications resulting in low utilization [26]. RFA is a novel alternative to PDT in the palliative approach to unresectable MBS. It involves administering thermal energy to strictures, leading to necrosis and cell death. The two RFA catheters in use are the Habib EndoHPB Bipolar Radiofrequency Catheter by Boston Scientific and the ELRA (Endoluminal Radiofrequency Ablation) by Taewoong Medical in South Korea [27]. These RFA catheters can be advanced over a guidewire towards the target lesion during ERCP (Figure 3).

There are two primary indications for RFA in patients with MBS, including palliative treatment of obstructive symptoms and ablation of SEMS tumor ingrowth. Kallis et al. found prolonged survival (228 vs. 124 days) in all-cause MBS patients who received RFA in combination with SEMS in comparison to SEMS alone [28]. Dolak et al. compared SEMS vs. PS in combination with RFA. They were unable to demonstrate a statistically significant difference in stent patency between SEMS and PS [27]. Sofi et al. performed a meta-analysis of 505 patients with biliary strictures and demonstrated improved survival and increased stent patency with RFA vs. non-RFA stents. However, in this study, RFA was associated with a higher risk of abdominal pain (31% vs. 20%). Other adverse effects of RFA include acute pancreatitis, cholangitis, cholecystitis, and hemobilia [29].

One challenging aspect of RFA is limiting ablation to healthy tissue surrounding the malignancy. A novel balloon catheter-based RFA system has been designed to solve this problem. Inoue et al. performed an experimental study using the balloon catheter-based RFA system and found that the accuracy of ablation was significantly improved, with a reduction in normal cell ablation [30]. In conclusion, RFA therapy significantly prolongs survival and improves stent patency for pancreatic cancer and cholangiocarcinoma. However, RCTs are needed to quantify the absolute survival benefit and determine the role of RFA in patients with unresectable HPB malignancy (Figure 3).

## 4. Endoscopic Ultrasound-Guided Biliary Drainage

Traditionally, when ERCP biliary drainage was not possible, percutaneous transhepatic biliary drainage (PTBD) was performed. PTBD was reserved for complicated cases, in which the duodenal papilla could not be accessed due to duodenal obstruction, abnormal postsurgical anatomy, or difficult cannulation. However, PTBD is a high-risk procedure, and newer methods like endoscopic ultrasound-guided biliary drainage (EUS-BD) are less-invasive and safer alternatives [31].

**Figure 4:**
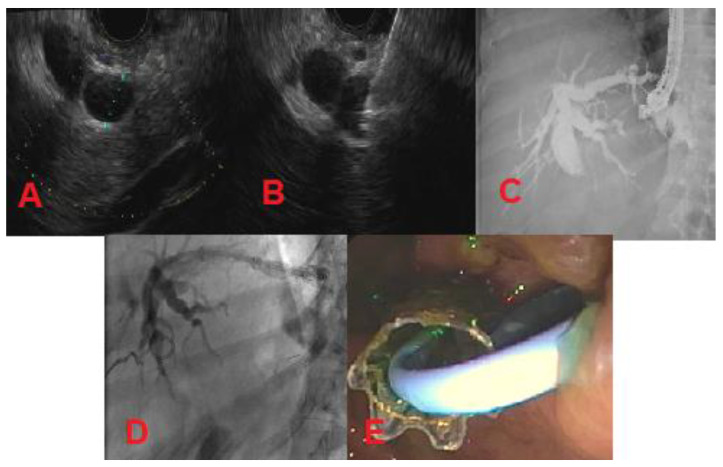
Metastatic pancreatic cancer (patient with history of Roux-en-Y gastric bypass surgery). (**A**) Dilated left intrahepatic biliary radicle; (**B**) Needle puncture of left intrahepatic biliary radicle using a 19-gauge needle; (**C**) Cholangiogram showing dilated intrahepatics, proximal and mid-common bile duct; (**D**,**E**) Left-sided hepaticogastrostomy.

There are three types of EUS-BD: intrahepatic, extrahepatic, and EUS-guided rendezvous (EUS-RV). The intrahepatic and extrahepatic methods are used when the duodenal papilla is not accessible. Intrahepatic EUS-BD involves puncture of a dilated left intrahepatic biliary radicle under EUS guidance through the lesser curvature of the stomach followed by tract dilation and antegrade stent placement (transpapillary) or placement of a metal stent between the stomach and the liver (EUS-guided hepaticogastrostomy) (Figure 4) [32]. Extrahepatic EUS-BD can be achieved for distal biliary obstructions by draining the dilated common bile duct under EUS guidance directly into the duodenum (EUS-guided choledochoduodenostomy) or by draining the distended gall bladder into the gastric antrum or duodenum in cases where the cystic duct is patent [33]. While the EUS-guided hepaticogastrostomy and choledochoduodenostomy methods bypass the stricture by forming an alternate route, EUS-RV aims to cannulate the stricture to increase forward flow of the bile. The rendezvous process involves identifying a dilated hepatic duct on EUS and placing a guidewire through the hepatic duct, past the stricture into the duodenum. At this point, a duodenoscope is used to cannulate the lesion over the guidewire to perform a conventional ERCP [31]. As described, there are multiple methods of EUS-BD. Currently, there is no standardized approach to choose one technique over another. More research is needed to characterize the risks and benefits of each procedure and its efficacy with various types of MBS. However, EUS-BD in general will likely replace PTBD as the field advances.

## 5. Conclusions

**Figure 5:**
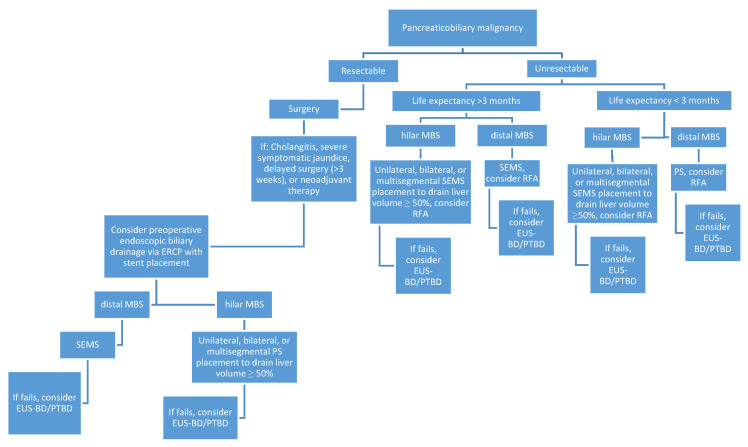
A flow chart summarizing the endoscopic approach to malignant biliary strictures.

The role of endoscopic management in malignant biliary strictures depends on lesion resectability and stricture location (Figure 5). Unfortunately, most lesions are unresectable at presentation and only palliative endoscopic techniques can be pursued. Palliative management has conventionally focused on biliary decompression via ERCP with stent placement. In instances where patients are not candidates for ERCP or the procedure is unsuccessful, endoscopic ultrasound-guided biliary drainage provides a reasonable alternative for biliary decompression. Biliary decompression will become an even more effective palliative tool with the development of new mesh materials and drug eluting stents which may improve stent patency. Endoscopic options are expanding past biliary drainage to include methods of tissue destruction like RFA and brachytherapy. The potential of these endoscopic innovations has yet to be fully determined as they are still in developmental stages, but they will redefine the approach to MBS patients who historically had few options.

## Figures and Tables

**Table 1:** Statistical comparison of Plastic stents (PS) vs. Self-expandable metallic stents (SEMS).

Meta-Analysis by Moole et al. Comparing PS vs. SEMS [12]				
	PS	SEMS	Complication	Odds Ratio SEMS vs. PS
Stent patency (days)	73.3	167.7	Cholangitis	0.46
Reintervention rate	1.7	1.1	Stent migration	0.45
Patient survival (days)	120.6	157.3	Cholecystitis	1.85
			Pancreatitis	0.80
			Occlusion rate	0.48

**Table 2:** Statistical comparison of self-expandable metallic stents: Fully-covered (FCSEMS) vs Uncovered (UCSEMS).

Meta-Analysis by Saleem et al. Comparing FCSEMS and USEMS for Distal MBS [17]			
	Weighted Mean Difference (Days) of FCSEMS vs. UCSEMS		Relative Risk (RR) of FCSEMS vs. UCSEMS
Patient survival	+51.2	Migration	8.11
Stent patency	+60.6	Overgrowth	2.03
Stent survival	+68.9	Sludge	2.89
		Ingrowth	0.23

**Table 3:** Bismuth–Corlette classification [22].

Type	Distinction
I	Limited to the common hepatic duct
II	Involving the confluence of the left and right hepatic ducts
IIIa	Involving the main hepatic confluence and extending to the bifurcation of the right hepatic duct
IIIb	Involving the main hepatic confluence and extending to the bifurcation of the left hepatic duct
IV	Involving the main, right, and left hepatic confluence
